# Case 1/2017 - Percutaneous Repair of Right Atrioventricular Valve Insufficiency and Blalock-Taussig Shunt after Fontan Operation in Single Ventricle

**DOI:** 10.5935/abc.20160201

**Published:** 2017-01

**Authors:** Edmar Atik, Renata Sá Cassar, Raul Arrieta

**Affiliations:** Clínica privada do Dr. Edmar Atik, São Paulo, SP - Brazil

**Keywords:** Heart Defects, Congenital / surgery, Mitral Valve Insufficiency / surgery, Blalock Taussig Procedure, Fontan Procedure

## Clinical data

Twenty-seven-year old male patient reporting tiredness during exercise for three years, after total cavopulmonary connection with extracardiac conduit fenestration and closure with stiches of the free end of right atrioventricular valve (AV) for severe failure in double inlet left ventricle, pulmonary atresia, and aorta arising from rudimentary right ventricle. The patient had undergone right and left Blalock-Taussig shunts at 17 days and 9 months, respectively, and bidirectional Glenn shunt at 19 years old. The patient had arterial oxygen saturation of 84-88% during exercise and 93% at rest.

Physical exam: eupneic, cyanotic, normal pulse rate, with no jugular vein dilation. Weight: 61 Kg, Height: 163 cm, BP: 110/70 mm Hg, HR: 97 bpm, oxygen saturation = 87%. The aorta was palpable in the supra-sternal notch (grade 2).

In the precordium, the apex beat was palpable at the fourth and fifth interspace and systolic impulses were slightly in the left sternal border (LSB). Accentuated heart sounds; grade 2 systolic murmur in the lower part and end of the LSB; continuous murmur was detected in pulmonary and axillary regions. The liver was not palpable.

### Complementary tests

**Electrocardiogram** showed sinus rhythm, signs of right atrial and ventricular overload. Peaked P waves in lead II, V3-6. QRS complex morphology with R wave amplitude of 10mm in V1 and RS in V6. QRS axis: +120º, T axis: +40º, PA: +55º.

**Figure 1:**
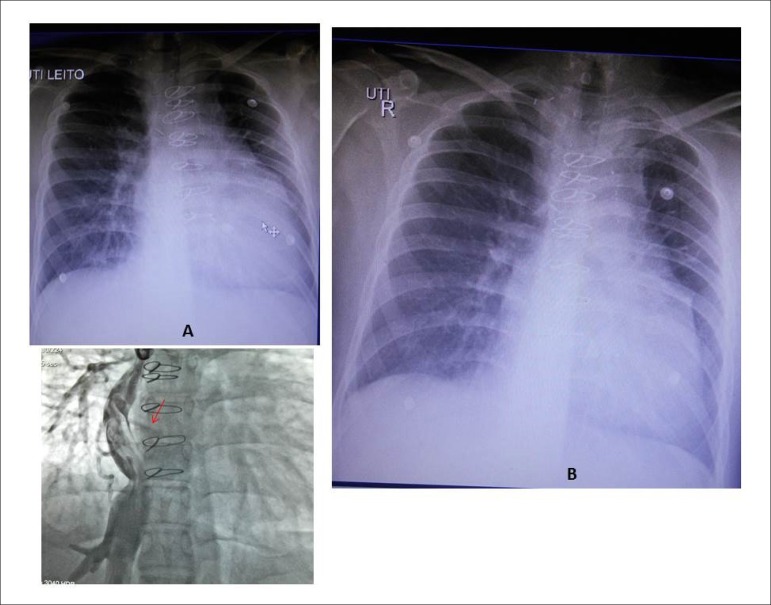
Chest radiographs showing increased heart area, elongated left ventricular and medial arches, and increased pulmonary vasculature before (A) and two days after percutaneous closure (B). The medial arch suggests the aorta arising from the left of the right ventricle. The angiography shows the connection between the inferior cava vein and right pulmonary artery with an extracardiac conduit by fenestrated total cavopulmonary connection (arrow).

**Chest radiograph** showed slight to moderate increase in heart area (cardiothoracic index: 0.54), elongated (left ventricular and medial) arches, and increased pulmonary vasculature (Figure 1).

**Figure 2:**
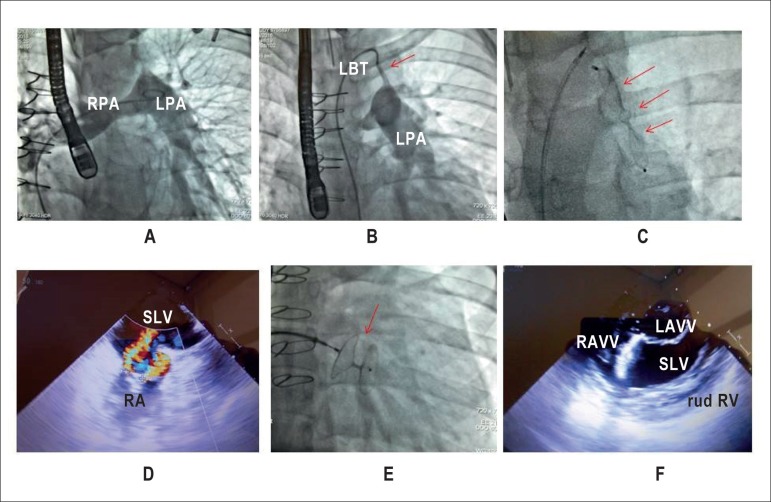
Cardiac angiography of pulmonary arterial tree showing the left pulmonary artery (LPA) pulled upwards (A), the Blalock-Taussig contrast medium in LPA (arrow) (B), and total occlusion of the Blalock-Taussig shunt using the Amplatzer device (C). The transesophageal echocardiography shows regurgitation of the right atrioventricular valve (RAVV) to the right (D), its occlusion by the Amplatzer device (E, F), and the wide left atrioventricular valve (LAVV) opening (F). RA: right atrium, RPA: right pulmonary artery, LPA: left pulmonary artery, LBT: left Blalock-Taussig, RAVV: right atrioventricular valve, LAVV: left atrioventricular valve, rud RV: rudimentar right ventricle, SLV: single left ventricle.

**Transesophageal echocardiography** (Figure 2) showed *situs solitus* and levocardia, systemic venous drainage at total cavopulmonary connection. Increased right atrium area and interatrial communication. Increased perimembranous ventricular septum in double inlet left ventricle, and aorta arising from a rudimentary chamber at left. Tricuspid annular plane systolic excursion (TAPSE): 9 mm. Ejection fraction calculated by the Simpson's rule method was 47%. Although the right AV had been surgically closed, there was a moderate regurgitation in the medial and anterolateral regions. Left AV with normal opening, and mild regurgitation. Pulmonary valve atresia with no Blalock-Taussig shunt.

**Cardiac catheterization** showed a mean pulmonary artery pressure of 15 mmHg with total cavopulmonary connection with the fenestrated tube (Figure 1), Blalock-Taussig shunt at left (Figure 2), and severe right atrioventricular regurgitation caused by medial and anterolateral paravalvular leak.

### Clinical diagnosis

Double inlet left ventricle, ventriculoarterial discordance and aorta at left, pulmonary atresia, left Blalock-Taussig shunt, cavopulmonary anastomosis with a fenestrated tube, paravalvular and right medial AV insufficiency, which was sutured during the Fontan procedure.

### Clinical reasoning

The clinical elements of cyanogenic heart diseases, as of left univentricular type and pulmonary hypoflow after total cavopulmonary are commonly innocent. Tiredness, systolic heart murmurs at the left sternal border and continuous murmur could be signs of right AV valve regurgitation, previously repaired, and continuation of the Blalock-Taussig shunt.

### Medical management

In light of the volumetric impact caused by the AV valvular insufficiency at right, in addition to deviation of blood flow through diversion through left Blalock-Taussig shunt, the repair of these residual lesions were found necessary. Since cardiac surgery with extracorporal circulation was considered of high risk, an Amplatzer septal occluder was placed in the right AV opening and closure of the left Blalock-Taussig shunt by percutaneous intervention. The right AV valve was closed using a 30-mm Amplatzer device and an Amplatzer duct occluder (ADO II, number 6) was used for the Blalock-Taussig shunt closure (Figure 2). The immediate recovery was satisfactory, with oxygen saturation greater than 90%, and a modest decrease in the cardiac area (Figure 1). The echocardiography revealed improved ventricular function (65%) with 4.5mm-fenestration, apparent continuous flow at Doppler and posterior desaturation rate of 82%-89%.

## Comments

Although the Fontan surgery is a palliative procedure that involves postoperative risk, it is still a promising approach if the indication criteria are strictly followed. In adults, due to acquired conditions related to heart diseases with long-standing overload, the operative risk is higher (10%). Within this context, the difficulty in establishing the surgical indication lies in acquired conditions such as ventricular dysfunction, anatomic valvular lesions, increased pulmonary pressure, among others. These factors should be counterbalanced with unfavorable clinical progress resulting from chronic hypoxia. In case the postoperative benefits overcome this, the clinical rationale should prioritize elements considered reversible. In the present case, the sutures made on the right AV valve during previous cavopulmonary surgery were removed; an Amplatzer device was placed and the Blalock-Taussig shunt was closed using the Amplatzer device at left. Therefore, it is expected that the patient makes a better progress after the repair of these residual lesions.

